# Sensor Distribution Design of Travel Time Tomography in Explosion

**DOI:** 10.3390/s140712687

**Published:** 2014-07-15

**Authors:** Yali Guo, Yan Han, Liming Wang, Linmao Liu

**Affiliations:** National Key Laboratory of Electronic Test Technology, North University of China, Taiyuan 030051, China; E-Mails: hanyan@nuc.edu.cn (Y.H.); wlm@nuc.edu.cn (L.W.); liulinmao@nuc.edu.cn (L.L.)

**Keywords:** sensor distribution design, travel time tomography, explosion, sub-region and multi-scale cells, adaptive escaping particle swarm optimization

## Abstract

Optimal sensor distribution in explosion testing is important in saving test costs and improving experiment efficiency. Aiming at travel time tomography in an explosion, an optimizing method in sensor distribution is proposed to improve the inversion stability. The influence factors of inversion stability are analyzed and the evaluating function on optimizing sensor distribution is proposed. This paper presents a sub-region and multi-scale cell partition method, according to the characteristics of a shock wave in an explosion. An adaptive escaping particle swarm optimization algorithm is employed to achieve the optimal sensor distribution. The experimental results demonstrate that optimal sensor distribution has improved both indexes and inversion stability.

## 1. Introduction

Explosion technology is applied increasingly [1,2]. As explosion experiments are complex and the costs are high, optimizing sensor distribution is necessary to save test costs and improve experiment performance. The number of sensors to be used and their locations should be optimized [3–5].

When a shock wave propagates in air, the shock wave velocity is very fast. Supposing that a shock wave propagates by direct rays without propagation by grid boundary, in the course of shock wave propagation, the relationship of travel time is:

(1) $$t=\int_{R}\frac{1}{v}\cdot dr=\int_{R}s\cdot dr$$

The wave front normal is defined as rays, *i.e.*, *r*. Each sensor corresponds to a ray. *v* is velocity and *s* is slowness. The travel time *t* is the integral of slowness along with rays.

**Figure 1.**
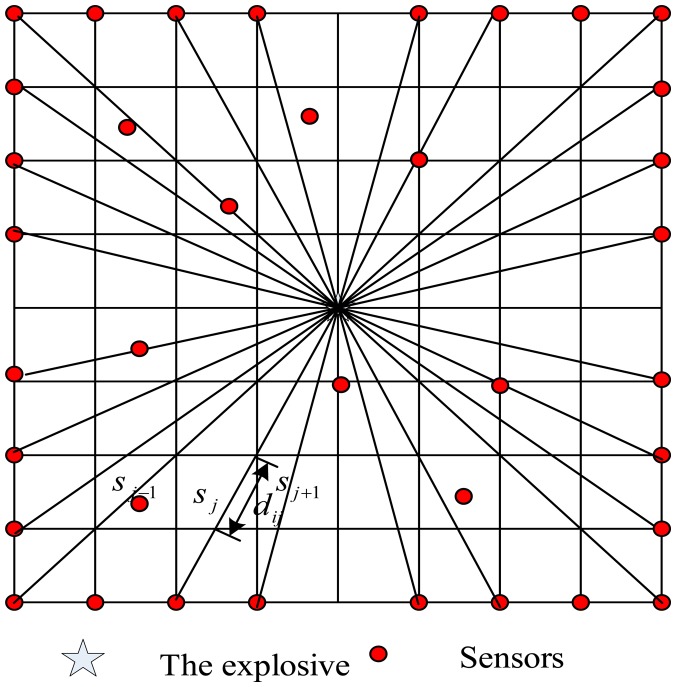
Illustration of travel time tomography.

The test region is divided into some regular or irregular cells as shown in Figure 1, the discretization expression of Equation (1) is:

(2) $$t_{i}=\sum_{j=1}^{N}d_{ij}s_{j}(i=1,2,3,\cdots ,M;j=1,2,\cdots ,N)$$

where *t_i_* is the travel time that a shock wave travels from the explosive to the *ith* sensor. *d_ij_* is the length of the *ith* ray in the *jth* cell. *S_j_* is the slowness in *jth* cell. M is the sensor number and N is the cell number. Equation (2) can be written in a matrix form as:

(3) DS=T

where *T* = (*t*_1_, *t*_2_, ⋯, *t_M_*)′ is the m-dimension column vector of travel time; *S* = (*s*_1_, *s*_2_, ⋯ *s_N_*)′ is an unknown *N* dimensional column vector; *D* is the distance matrix of *M*× *N* and its element is *d_ij_* [6,7]. The elements of *T* can be obtained by the tested data from sensors. The matrix *D* can be calculated by the position of the explosive and sensors. The unknown slowness *S* can be obtained by solving Equation (3). Based on the above-mentioned principle, the velocity distribution of shock wave can be obtained.

For the travel time tomography, the driving source is single and the sensors are few. The tomography rays are distributed sparsely. The Equation (3) is ill-posed and underdetermined [8]. For this tomography modality, optimizing sensor distribution is necessary for reducing costs and increasing acquired information. Optimizing sensor distribution is to solve ill-posed tomography problems from the perspective of mathematics and then the inversion stability can be ensured.

The ill-posed degree of Equation (3) is related to the structure of matrix *D*. Improving the ill-posed degree of Equation (3) is to improve the structure of matrix *D*. The structure of matrix *D* rests with a parameterized model of the test region and sensor distribution [9].

## 2. Theory of Optimal Sensor Distribution and Indexes

### 2.1. Effect of Eigenvalue and Rank on Inversion

In Equation (3), with a given data vector *t*_0_, the model vector *s*_0_ ∈S and then |*t*_0_ − *Ds*_0_|^2^ is minimized. This is accomplished by pre-multiplying Equation (3) by *D^T^* and taking a matrix inverse:

(4) $$S={\left(D^{T}D\right)}^{-1}D^{T}T$$

As the *N* ×*N* square matrix *L*=*D^T^D* is often near-singular, it results in instability in the solution. That is, some of its eigenvectors {*e_i_*: *i*=1,⋯,*N*} have extremely small eigenvalues {*λ_i_*:*i*=1,⋯,*N*}. Measurement errors in data space T propagate into the solution *S* in parallel to each eigenvector with an amplification 1/λ*_i_*. Hence, when small eigenvalues exist, the solution becomes unstable and the inverse problem is ill-posed [10]. When an eigenvalue is zero, the corresponding eigenvector in the data space can not be mapped into the model space [11]. The greater the rank is and the larger the eigenvalues are, the more stable the inversion problem is and the more independent pieces of information may be gained from the data. Hence, optimal sensor distribution can be obtained by maximizing the magnitude of eigenvalues of *L* and the rank of *D*. The evaluating function is:

(5) $$E_{1}=\frac{N\lambda_{1}}{\text{trace}(D^{T}D)}+N-\text{rank}(D)$$

where λ_1_ is the maximal eigenvalues of *D_T_D*; $\text{trace}\left(D^{T}D\right)=\overset{N}{\underset{i=1}{\text{∑}}}\lambda_{i}$ ; *_N_* is the cells number, *rank*(*D*) is the rank of matrix *_D_*.

The evaluating function *E*_1_ has two components, one represents the relative distribution of eigenvalues and the other indicates the relative size of null space. If the value of *E*_1_ is minimal, the inversion results are optimal and stable.

### 2.2. Effect of Condition Number on Inversion

The Equation (3) is ill-posed if small perturbations of matrix *D* or T can result in a large change in solutions [12].

Assuming that the observed data T has a minor perturbation of *δT*, the perturbation of solution is *δS*. Equation (3) becomes:

(6) D(S+δS)=T+δT

Then,

(7) $$\delta S=D^{-1}\delta T$$

According to the property of subordinate norm, ‖*δ*S‖=‖*D*^−1^*δT*‖≤ ‖*D*^−1^‖‖*δT*‖ and $\left\|S\right\|\geq \frac{\left\|DS\right\|}{\left\|D\right\|}=\frac{\left\|T\right\|}{\left\|D\right\|}$. It concludes that $\frac{\left\|\delta S\right\|}{\left\|S\right\|}\leq \frac{\left\|D^{-1}\right\|\left\|\delta T\right\|}{\left\|T\right\|/\left\|D\right\|}$. That is $\frac{\left\|\delta S\right\|}{\left\|S\right\|}\leq \text{cond}\left(D\right)\frac{\left\|\delta T\right\|}{\left\|T\right\|}.$

Then,

(8) $$\text{cond}(D)=\left\|D\right\|\left\|D^{-1}\right\|$$

where *cond*(*D*) is the condition number of matrix *D*. When the observed data *T* has a minor perturbation, the relative error of solution is determined by the condition number.

Supposing that T is accurate and the matrix *D* has a minor perturbation of *δ_D_*, the corresponding perturbation of solution is *δS*. Supposing that *S _c_* = *S* + *δS* is the solution of perturbation equation,

(9) $$(D+\delta D)\cdot S_{c}=(D+\delta D)\cdot (S+\delta S)=T$$

Similarly the following condition can be obtained:

(10) $$\frac{\left\|\delta S\right\|}{\left\|S+\delta S\right\|}\leq \text{cond}(D)\frac{\left\|\delta D\right\|}{\left\|D\right\|}$$

When the matrix *D* has a minor perturbation, the error of solution is also determined by the condition number.

Therefore, the condition number is the second judging index of matrix *D*'s quality. The smaller condition number means the more well-posed equation.

The evaluating function expressed by the condition number can be written as:

(11) $$E_{2}=\text{cond}(D)$$

Optimal sensor distribution can reduce the condition number and the more stable inversion solutions can be obtained.

### 2.3. Effect of Ray Coverage on Inversion

When designing sensor location, rays coverage should be enlarged as much as possible, *i.e.*, rays density and orthogonality should be maximized. The ray density represents the number of rays passing through each cell. The cells not being hit by any ray are the main factors giving rise to the null space. They make some column vectors of matrix *D* be zero (*d_j_* = 0) so that the equation can not be resolved. Therefore, tomography with sparse rays must ensure that any column vector is non-zero. Increasing the ray density can avoid zero vectors.

Ray orthogonality is measured by maximal sinusoidal quantity of angle between rays [13]. The small orthogonality makes some rows in *D* linearly dependent.

The greater the ray density is, the better the orthogonality is and the smaller inversion error can be achieved. The evaluating function expressed by rays density and orthogonality can be written as:

(12) $$E_{3}=\frac{k_{1}}{N}\sum_{j=1}^{N}\rho_{j}+\frac{k_{2}}{N}\sum_{j=1}^{N}O_{j}=k_{1}\bar{\rho }+k_{2}\bar{O}$$

where *ρ_j_* is the ray density in the *jth* cell. *O_j_* is the ray orthogonality in the *jth* cell. The value of *k*_1_ and *k*_2_ are determined by the values of *ρ_j_* and *O_j_*. Sensor distribution can be optimized by maximizing *E*_3_.

### 2.4. Evaluation Method

As sensor distribution can be optimized by minimizing *E*_1_ and *E*_2_ and maximizing *E*_3_, an evaluating function is defined as:

(13) $$E=\omega_{1}E_{1}+\omega_{2}E_{2}+\frac{\omega_{3}}{E_{3}}$$

where ω_1_, ω_2_, and ω_3_ are determined by the values of *E*_1_, *E*_2_, and *E*_3_. Sensor distribution can be optimized in terms of minimizing *E*.

**Figure 2.**
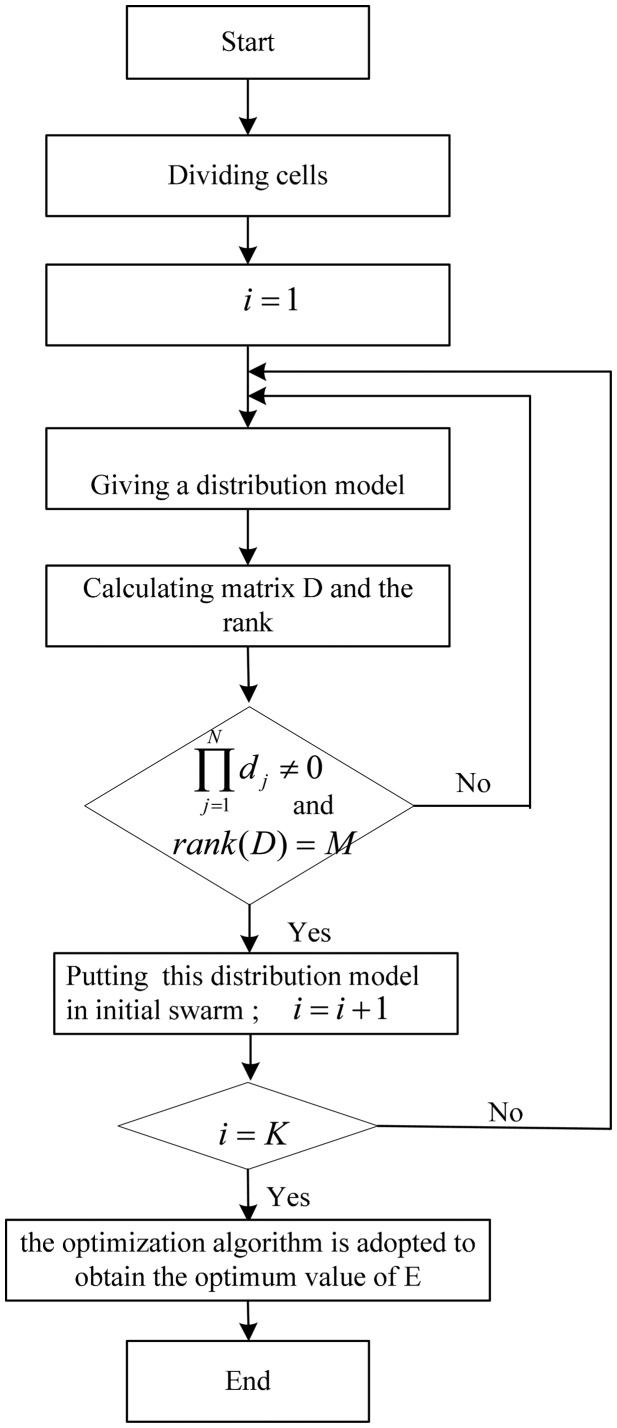
Illustration of evaluation process.

There are many factors on the ill-posed and underdetermined equations except for the above analysis. However, the value of *_E_* can be used as a judging index with regard to optimizing sensor distribution and the experiment. The detailed process is as follows and a flow chart is illustrated in Figure 2.

(1) Dividing cells according to model character.
(2) Giving a distribution model randomly according to the number of sensors and calculating matrix *D* and the rank of *D*.
(3) If all column vectors of matrix *D* are non-zero and *D* is full rank, make this distribution model as an initial model; otherwise, go to Step (2).
(4) When the number of initial model is equal to the given number *K*, the optimization algorithm is employed to obtain the optimum value of E and sensor distribution.

## 3. Optimizing Sensor Distribution

### 3.1. Sub-Region and Multi-Scale Cells Partition

The test region is divided into cells and each cell has the same velocity. The dividing pattern of cells affects matrix *D*. We propose a sub-region and multi-scale cell partition method. Cells are divided in terms of the solution distribution, *i.e.*, the smaller size and the higher density of cells correspond to the bigger changing gradient of solutions, and the bigger size and the lower density correspond to the smaller changing of solutions.

When the explosive is exploding in air, the shock wave overpressure attenuates quickly in the near region to the explosive and the attenuation becomes slow with the increase in distance. According to the shock wave characteristic, the smaller cells are adopted in the near region to the explosive while the bigger cells are adopted in the far region. In order to avoid a row vector to be linearity dependent on matrix *D* aroused by symmetry, cells are divided into different size in the symmetrical region with consideration of the resolution of inversion.

### 3.2. Optimizing Sensor Distribution Based on Adaptive Escaping Particle Swarm Optimization Algorithm

#### 3.2.1. Particle Swarm Optimization (PSO) Algorithm and Modification

Particle swarm optimization (PSO) algorithm is simple and easy to implement. However, PSO can fall into the local optimum [14,15]. The original algorithm is modified and an adaptive escaping particle swarm optimization algorithm (AEPSO) is proposed.

Supposing that *X_i_* = (*x_i_*_1_, *x_i_*_2_,⋯, *x_id_*) is the present position of *ith* particle; *V_i_* = (*v_i_*_1_, *v_i_*_2_,⋯, *v_id_*) is the present flying speed of *ith* particle; *P_i_* = (*p_i_*_1_, *p_i_*_2_,⋯, *p_id_*) is the individual optimal fitness of *ith* particle; *P_g_* = (*p_g_*_1_, *p_g_*_2_,⋯, *p_gd_*) is group optimal fitness; *d* is particle dimension, the evolution equations of original PSO algorithm are:

(14) $$v_{i}^{k+1}=\omega v_{i}^{k}+c_{1}r_{1}(p_{i}^{k}-x_{i}^{k})+c_{2}r_{2}(p_{g}^{k}-x_{i}^{k})$$

(15) $$x_{i}^{k+1}=x_{i}^{k}+v_{i}^{k+1}$$

where, *k* is iteration times. *ω* is inertia weight; *c*_1_ and *c*_2_ are the random numbers between (0, 2) and called learning factors; *r*_1_ and *r*_2_ are the random numbers between (0, 1) [16,17].

When *P_g_* does not change over M generations, all particles are close to *P_g_*. The flying speed of particles is very little and subsistence density is large. An escape strategy should be adopted to update the velocity of particles and enlarge the searching space. The escape strategy changes the velocity of particles and makes variation in order to increase the diversity of particles and jump out of local optimization. When escaping, the particles are divided into two components, one component updates their velocity according to Equation (16) and the other component updates their velocity according to Equation (17).

(16) $$v_{id}^{k+1}=r_{3}\;\cdot v_{id}^{k}\;\cdot \;(A-v_{id}^{k})'$$

(17) $$v_{id}^{k+1}=r_{4}\;\cdot \;v_{\max}$$

where *r*_3_ and *r*_4_ are the random numbers between (0, 1). *A* is a constant that controls the particle velocity. *v*_max_ is the maximal velocity value that particles have.

The declining linearly inertia weight helps to swarm searching. The adjusting strategy is as follows:

(18) $$w(k)=w_{m,ax}-\frac{\left(w_{\max}-w_{\min}\right)}{q_{\max}}\times k$$

where *w*(*k*) is the current inertia weight; *w*_max_ and *w*_min_ are the maximum and minimum inertia weights; *q*_max_ is the maximum number of iterations; *k* is current iteration times.

#### 3.2.2. Optimizing Sensor Distribution Based on AEPSO Algorithm

**Figure 3.**
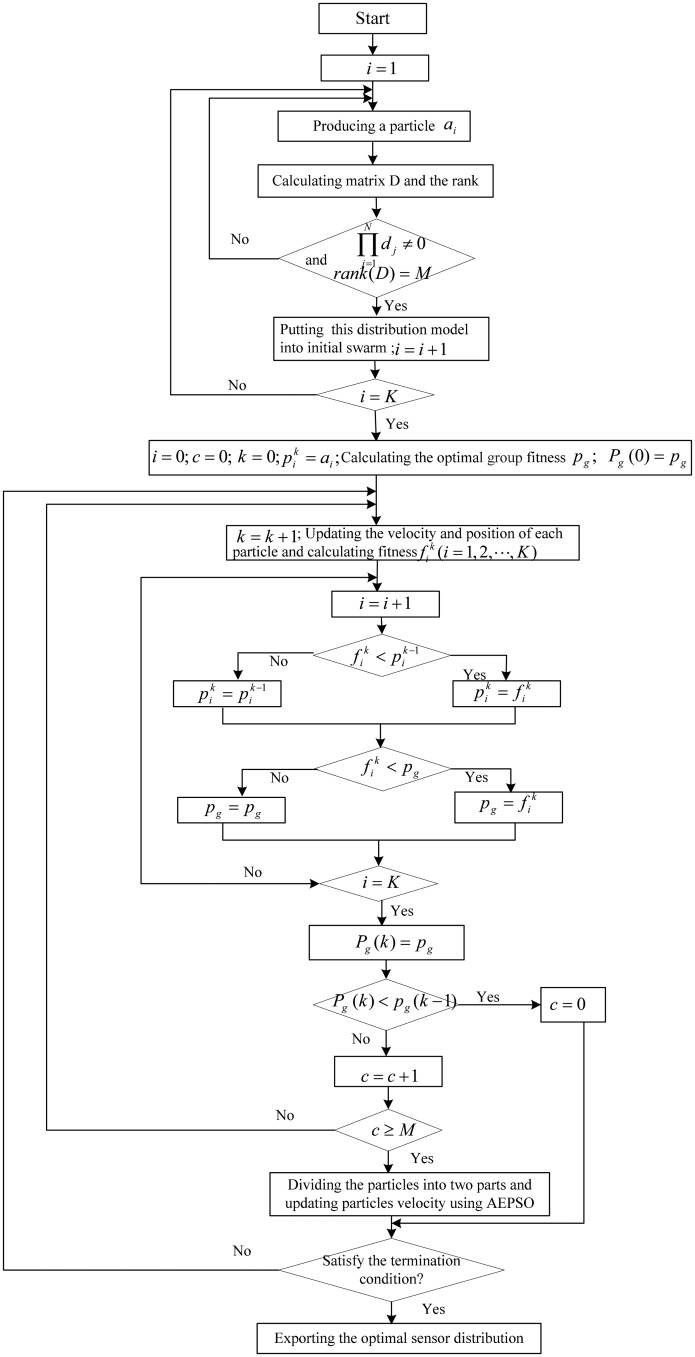
Process of optimizing sensor distribution.

The adaptive escaping particle swarm optimization algorithm is adopted to obtain the optimum value of *E* and the sensor distribution. The detailed process is as follows and a flow chart is illustrated in Figure 3.

(1) Producing a particle. A d-dimension particle is produced by selecting a distribution model randomly according to the number of sensors, which is expressed as *a* = (*xr*_1_, ⋯, *xr_m_* , *yr*_1_, ⋯, *yr_m_*), where *xr_j_* and *yr_j_* represent *x* and *y* coordinate of *jth* sensor, respectively, and *m* is the number of sensors; *d* = 2*m*.
(2) Calculating matrix *D* and the rank of *D*.
(3) If all column vectors of matrix *D* are not zero and matrix *D* is full rank, put this particle into the initial particle swarm; otherwise, return to Step (1).
(4) Calculating the optimal group fitness when the number of particles in initial swarm is equal to the given number.
(5) Updating the velocity and position of each particle according to Equations (14) and (15) and calculating fitness.
(6) Updating the individual optimal fitness. If the current fitness is superior to experienced optimal fitness *P_i_*, the experienced optimal fitness *P_i_* is replaced by the current fitness.
(7) Updating the group optimal fitness. If the current fitness is superior to the group optimal fitness, the group optimal fitness *P_g_* is replaced by current fitness.
(8) Recording group optimal fitness *P_g_* in each iteration. If the value of *P_g_* does not change over *M* generations, return to Step (9) and adopt escaping strategy; otherwise, return to Step (5).
(9) Dividing the particles into two components and updating particles velocity according to Equations (16) and (17).
(10) If the ending condition is met, terminate the iteration or return to Step (5).

## 4. Numerical Simulations

### 4.1. Comparison of Cell Performance

**Figure 4.**
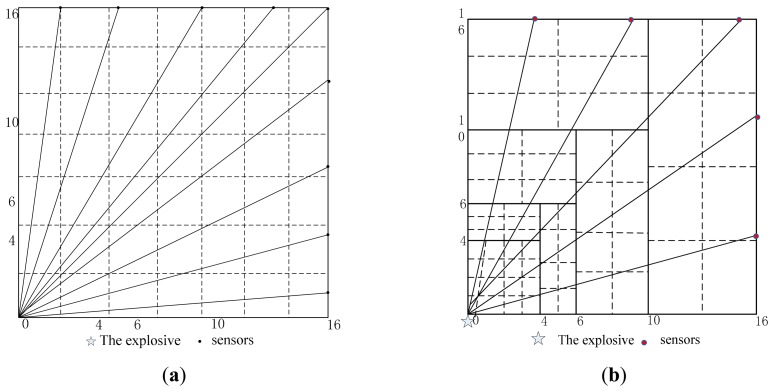
Two cell patterns. (**a**) Uniform rectangle cells; (**b**) Multi-scale cells.

To verify the sensor distribution design, a test region is 16 m × 16 m, explosive is placed in the lower-left and sensors are distributed on the region boundary. Two cell patterns are used in dividing the test region. The first one is a uniform rectangle with 49 cells as shown in Figure 4a. The second one is sub-region and multi-scale cells as shown in Figure 4b. The velocity of shock wave decreases exponentially with the distance according to the prior information. In the near region to the explosive, the attenuation amplitude of velocity is larger and the cells are smaller and denser. In the far region the attenuation amplitude of velocity is smaller and the cells are bigger and sparser. The region is divided into seven sub-regions according to the proportional distance and different size cells in the symmetry region are placed in order to avoid a row vector to be linearity dependent on matrix *D*. The total cells number is 58.

The influence of cells on matrix *D* is compared with the same sensor distribution.

(1) To ensure each cell being passed through by at least one ray, the lowest number of sensor required by uniform rectangle cells is 9 as shown in Figure 4a. The multi-scale cells require at least five sensors as shown in Figure 4b.
(2) Each index of matrix *D* in uniform rectangle cells and multi-scale cells is compared with 13 sensors and the same distribution. Simulation results are given in Table 1. It is clear that the indexes in multi-scale cells are superior to those of the uniform rectangle cells. The matrix *D* in multi-scale cells is full rank mostly and the condition number is far smaller than that of uniform rectangle cells. The ray density and orthogonality in multi-scale cells are larger than that of uniform rectangle cells generally.

### 4.2. Optimizing Sensor Distribution Based on Multi-Scale Cells

#### 4.2.1. Parameters Setting and Simulation of AEPSO Algorithm

To validate the AEPSO algorithm, two multi-modal functions are used to test the PSO and AEPSO algorithms.

Rastrigrin function:

(19) $$f_{1}(x)=\sum_{i=1}^{d}\left[x_{i}^{2}-10\cos(2\pi x_{i})+10\right](-10\leq x_{i}\leq 10)$$

Griewank function:

(20) $$f_{2}(x)=\frac{1}{4000}\sum_{i=1}^{d}x_{i}^{2}-\prod_{i=1}^{d}\cos(x_{i}/\sqrt{i})+1(-500\leq x_{i}\leq 500)$$

The two functions have many local minimum values, which has the global minimum value of 0 when *x_i_* = 0. The mean best fitness (MBF) and standard deviation (SD) are used to estimate the solutions. Parameters setting are given in Table 2.

**T**he simulation results are given in Table 3. From the table, it is concluded that AEPSO is superior to PSO algorithm in accuracy, convergence, and stability.

#### 4.2.2. Optimizing Sensor Distribution

**Figure 5.**
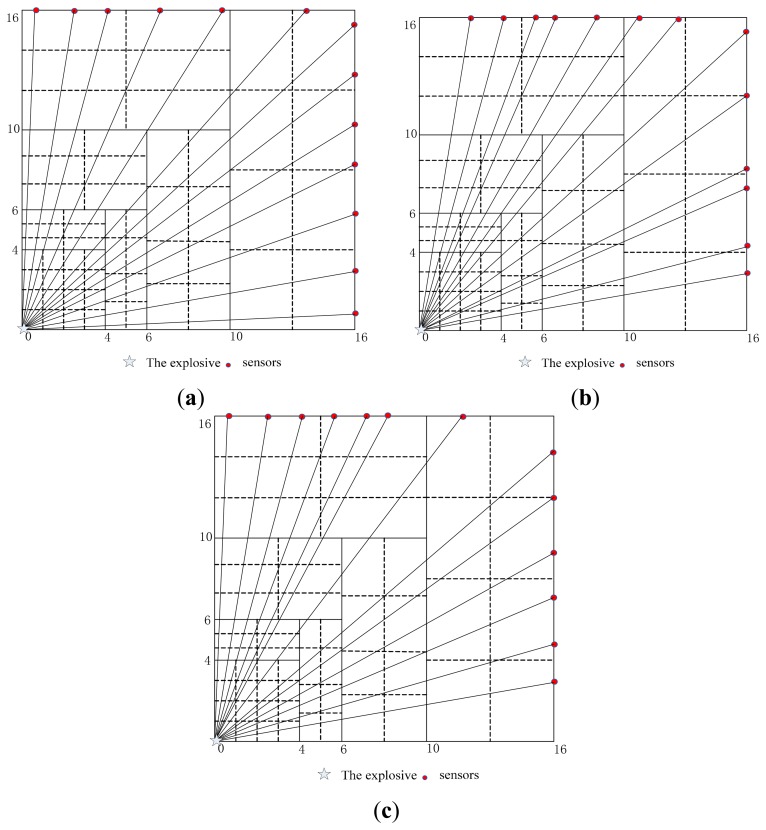
Different sensor distributions, respectively in each case. (**a**) Symmetrical sensor distribution; (**b**) Random sensor distribution; (**c**) Optimum sensor distribution.

**Figure 6.**
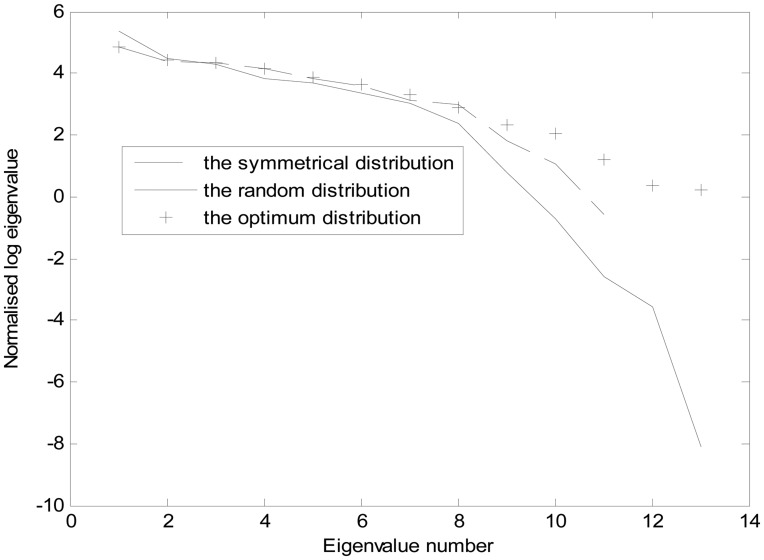
Comparison of eigenvalue spectra.

Each index based on multi-scale cells with symmetrical distribution and random distribution of sensors is shown in Figure 5a,b. According to the values of *E*_1_, *E*_2_, *ρ̅* and *O̅*, the weight coefficients are selected as: *ω*_1_ = 0.8, *ω*_2_ = 0.2, *ω*_3_ = 15, *k*_1_ = 0.1, *k*_2_ = 0.9. The adaptive escaping particle swarm optimization (PSO) algorithm is adopted to obtain the optimum value of *E* and sensor distribution as shown in Figure 5c. The values of *E*_1_, *E*_2_, *ρ̅* and *O̅* with different sensor distributions are in Table 4. It is clear that the indexes in optimum distribution are superior to that of the others: smallest *E*_1_ and *E*_2_, and the biggest *E*_3_. Figure 6 shows a comparison of the normalized eigenvalue spectra respectively in each case. The optimized model is again superior to that of the others. Numerical simulations are presented with the same initial model and inversion algorithm. From the results it can be concluded that the optimum distribution can result in the smallest inversion error.

## 5. Conclusions

For travel time tomography in explosion, optimizing sensor distribution is very important for saving the costs and increasing the acquired information. This paper shows the inversion stability can be improved by designing optimal sensor distribution. This paper analyzed the effect of eigenvalue, rank, condition number and rays coverage on improving matrix *D* and proposes the evaluating function on optimizing sensor distribution. An adaptive escaping particle swarm optimization algorithm is used to obtain the optimum sensor distribution based on sub-region and multi-scale cells. Simulation shows that the optimization method is feasible and the optimal sensor design achieves inversion stability.

## Figures and Tables

**Table 1.** Each index and simulation results.

**Cells Pattern**	**Number**	**Rank**	**E1**	**E2**	*O̅*	*ρ̅*
**Multi-Scale Cells**	1	13	53.08	111.08	0.220	3.334
	2	13	55.38	79.71	0.225	3.235
	3	13	55.57	50.38	0.229	3.235
	4	13	56.04	3285.90	0.227	3.353
	5	13	57.34	1178.80	0.185	3.235
	6	13	59.32	834.00	0.206	3.353
	7	13	59.73	972.40	0.218	3.451

**Uniform Rectangle Cells**	1	12	54.49	1.76 × 10^16^	0.134	2.653
	2	12	56.51	6.89 × 10^15^	0.149	2.755
	3	12	56.87	1.02 × 10^16^	0.145	2.694
	4	12	57.58	9.45 × 10^15^	0.151	2.755
	5	12	58.54	8.86 × 10^15^	0.139	2.857
	6	11	60.91	1.24 × 10^16^	0.141	2.857
	7	11	60.38	2.14 × 10^16^	0.141	2.898

**Table 2.** Parameters setting.

**Algorithm**	*ω*	*c*_1_	*c*_2_	**A**	**M**
PSO	0.7	1.49	1.49	_	_
AEPSO	[0.9, 0.4]	1.49	1.49	1	10

**Table 3.** Simulation results.

**Function**	**Dimension**	**Algorithm**	**MBF**	**SD**
Rastrigrin Function	10	PSO	40.1	27.4
		AEPSO	1.8 × 10^−2^	2.1 × 10^−2^

	30	PSO	149.7	45.3
		AEPSO	8.5 × 10^−2^	1.4 × 10^−1^

Griewank Function	10	PSO	2.6 × 10^−1^	1.2 × 10^−1^
		AEPSO	2.5 × 10^−4^	6.9 × 10^−4^

	30	PSO	6.8	7.1
		AEPSO	3.6 × 10^−2^	4.5 × 10^−2^

**Table 4.** Comparison of each index in different distributions.

**Sensor Distribution**	**Symmetrical Distribution**	**Random Distribution**	**Optimum Distribution**
Rank	11	13	13
E1	56.68	58.90	50.84
E2	3.67 × 10^16^	887.70	10.20
E3	0.48	0.51	0.57
*_O̅_*	0.178	0.190	0.254
*_ρ̅_*	3.157	3.373	3.373
E	7.35 × 10^15^	254.16	69.24
Related error (%)	7.89	7.41	5.51
